# Observed and predicted premature mortality in Australia due to non-communicable diseases: a population-based study examining progress towards the WHO 25X25 goal

**DOI:** 10.1186/s12916-022-02253-z

**Published:** 2022-02-10

**Authors:** Alison Wijnen, Karen Bishop, Grace Joshy, Yuehan Zhang, Emily Banks, Ellie Paige

**Affiliations:** 1 National Centre for Epidemiology and Population Health, Australian National University, Canberra, Australia; 2 The Sax Institute, Sydney, Australia

**Keywords:** Non-communicable diseases, Cancer, Cardiovascular disease, Chronic respiratory diseases, Diabetes, WHO 25X25 goal, Premature mortality, Sociodemographic factors

## Abstract

**Background:**

The World Health Organization’s (WHO) 25X25 goal aims for a 25% relative reduction in premature death due to four non-communicable diseases (NCD4)—cancer, cardiovascular disease, chronic respiratory diseases and diabetes—by 2025 compared to 2010. This study aimed to quantify the premature mortality in the Australian population due to NCD4, quantify the variation in mortality rates by age and sex, predict the premature mortality due to NCD4 in 2025 and evaluate the progress towards the WHO 25X25 goal.

**Methods:**

A population-based study using cause-specific mortality data of all deaths which occurred in Australia from 2010 to 2016 and registered up to 2017, for adults aged 30–69 years, was conducted. Age-specific and age-standardised mortality rates (ASMR) and probability of death for NCD4 were calculated for each year. ASMRs in 2016 were calculated for men and women. Deaths and the probability of death in 2025 were predicted using Poisson regression based on data from 2006 to 2016. To assess the progress against the WHO 25X25 goal, the relative reduction in the probability of death from NCD4 conditions in 2025 compared to 2010 was calculated.

**Results:**

ASMRs for NCD4 decreased from 2010 to 2016, except for diabetes which increased on average by 2.5% per year. Across sociodemographic factors, ASMRs were highest in males and increased with age. The projected probability of premature death in 2025 was 7.36%, equivalent to a relative reduction of 25.16% compared to 2010 levels.

**Conclusions:**

Premature mortality due to cancer, cardiovascular disease, respiratory diseases and diabetes declined in Australia from 2010 to 2016. This trend is consistent across age groups and by sex, and higher mortality rates were observed in males and at older ages. Nationally, if the current trends continue, we estimate that Australia will achieve a 25.16% relative reduction in premature deaths due to NCD4 in 2025 compared to 2010, signifying substantial progress towards the WHO 25X25 goal. Concerted efforts will need to continue to meet the 25X25 goal, especially in the context of the COVID-19 pandemic.

**Supplementary Information:**

The online version contains supplementary material available at 10.1186/s12916-022-02253-z.

## Background

Non-communicable diseases (NCDs) cause around 15 million deaths annually worldwide, in people aged 30–69 years, most of which are preventable with changes to modifiable risk factors [1]. NCDs are responsible for over 70% of deaths globally and 89% of deaths in Australia [2]. In Australia, NCDs comprise the majority of the burden of disease with cancer being the largest contributor, followed by cardiovascular diseases (CVD), with respiratory diseases and endocrine diseases, including diabetes, being the 6th and 9th largest contributors, respectively, in 2015 [3].

In 2013, member states of the World Health Organization (WHO), including Australia, agreed to adopt a global framework for monitoring progress towards decreasing mortality from NCDs [4]. The NCD monitoring framework has nine voluntary targets for 2025, including a target to reduce premature mortality from four NCDs (hereafter referred to as NCD4) by 25% compared to 2010 levels: known as the 25X25 goal. Progress towards the WHO 25X25 goal is measured by a relative reduction in the probability of death from four major NCDs—cancer, CVD, chronic respiratory diseases and diabetes—in people aged 30–69 years inclusive [5].

A number of studies have been published considering progress towards the 25X25 goal and the related 30 by 2030 sustainable development goal (SDG) which aims for a 30% relative reduction in the probability of premature death by 2030 compared to 2015 levels. These indicate that WHO methodology and modelling can be used to measure premature mortality and evaluate the progress towards the goal. A Swedish study showed that a 25% relative reduction in premature mortality due to NCDs had been achieved in the period 1991 to 2006 [6]. They concluded that further reductions to achieve the 25X25 goal may be challenging in Sweden and other countries with similarly low mortality rates [6]. A UK study modelled future population, mortality and population exposure to risk factors and showed that reducing levels of obesity would have the greatest reduction on expected mortality in 2025 [7]. A study conducted in China examined the progress towards the 2030 SDG [8]. They found that the target would not be met due to expected trends in cancer and diabetes mortality [8]. The NCD Countdown Collaborators took a similar approach to examine the 2030 SDG and found the lowest probability of premature death in 2015 was in high-income countries including Australia. Their modelling indicated that for Australia to meet the 2030 goal, a faster rate of decline would be needed in several disease groups including chronic respiratory diseases [9].

A recent WHO report on international progress towards the NCD monitoring framework goals showed that in 2016 the probability of NCD premature death for Australia was low in comparison with international standards, at 9.0%. This represents a 9.2% relative reduction compared to the probability of 9.9% reported in 2010 [10]. NCD Countdown Collaborators [11] have modelled the probability of premature death up to 2030. However, there are no published projections on the expected relative decrease in NCD deaths in Australia by 2025 specifically or investigation of the relative contribution of the individual NCDs towards meeting the WHO 25X25 goal. There is also limited data on how mortality due to NCD4 varies in Australia by age and sex. Such data are important not only for identifying whether Australia is likely to meet the WHO 25X25 goal based on current trends, but for identifying possible points to intervene to further reduce mortality from NCD4 across the population.

The aims of this study are to (1) quantify premature mortality in the Australian population from 2010 to 2016 that is due to the four major NCDs: cancer, CVD, chronic respiratory diseases and diabetes, separately and combined; (2) quantify variation in premature NCD4 mortality rates by age and sex; (3) predict expected premature mortality for the NCD4 separately and combined in 2025; and (4) compare progress against the WHO 25X25 goal target probability of dying from one of the NCD4 conditions.

## Methods

### Study population

This study is based on adults resident in Australia aged 30–69 years in 2010–2016 using data on the Estimated Resident Population for Australia [12].

### Data sources

Deaths registered in Australia from 2006 to 2016 for the study population were obtained from the Cause of Death Unit Record Files provided by the Australian Registrars of Births, Deaths and Marriages and the Coroners and the National Coronial Information System. Australian mortality data have a five-star rating (highest possible) for data quality, including coverage of death registrations, from the Global Burden of Disease study. Across all ages for the years of interest, there were only 126 records with age not stated. Several different sources of information are used to identify sex when not stated, resulting in no missing data by sex for the mortality data used in this study.

The Estimated Resident Population for Australia and projected Australian populations were obtained from the Australian Bureau of Statistics. The Australian Bureau of Statistics provides three alternate population projection scenarios, series A, B and C, for Australia [13]. Series B reflects current trends in migration, fertility and life expectancy, while series A and C predict the expected Australian populations under an increase and decrease, respectively, in trends of migration, fertility and life expectancy. Series B population projection scenarios are used in the main analysis and series A and C are used in sensitivity analyses (details below).

### Outcome

The main outcome was death from one of NCD4, ascertained from the underlying cause of death in the Cause of Death Unit Record Files. The International Classification of Diseases 10, Australian Modification (ICD-10-AM) codes were used to identify deaths due to cancer (C00–C97), CVD (I00–I99), chronic respiratory diseases (J30–J98) and diabetes (E10–E14) [14, 15].

### Statistical analysis

#### Observed deaths, death rates and probability of dying

Measures were calculated using 5-year age and sex groups, for each NCD4 separately (CVD, cancer, chronic respiratory diseases, diabetes) and combined.

Age-standardised mortality rates (ASMRs) for cancer, CVD, chronic respiratory diseases and diabetes were calculated separately and combined for the population aged 30–69, for each year 2010–2016, using direct standardisation to the 2011 Estimated Resident Population of Australia [12]. Death records with missing age at death were excluded (*n* = 83).

The annual percentage change in ASMRs was calculated for each year in the period 2010–2016 and averaged over this period.

Using the life table method recommended for the WHO 25X25 indicator (Table 1), we calculated the annual age-specific probability of premature death in 5-year age groups for people aged 30–69 years and each NCD4 condition, over the period 2010–2016 [14].

**Table 1:**
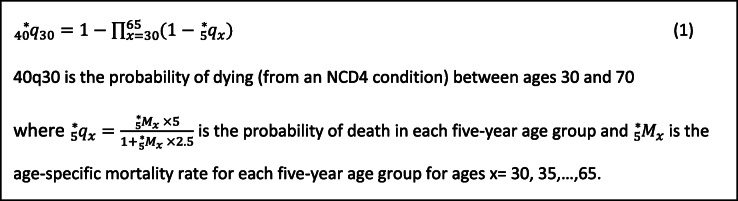
Life table method for calculating the unconditional probability of death

#### Projected deaths, death rates and probability of dying

Poisson regression with a log-linear link function was used to predict the number of NCD4 deaths in each year from 2017 to 2025 by sex and 5-year age groups. Poisson regression was used as the outcome modelled was count data (number of deaths), and log-linear link function was used to ensure that modelled decreasing trends in mortality would not fall below zero. The Poisson models included the observed annual number of deaths in each 5-year age and sex group using all available data from 2006 to 2016 as the dependent variable, time/year as the independent variable and an offset term was included as the exposure time represented by the Estimated Resident Population in the corresponding year of projected population series B, 2017-2025. The model predicted the number of NCD4 deaths which were then used with the projected populations for Australia from 2017 to 2025 to calculate annual age-specific and age-standardised mortality rates for each NCD4 condition through to 2025. We used series B population projections for the main analysis to predict deaths on current trends in migration, fertility and life expectancy.

The projected age-specific mortality rates were used to estimate the probability of premature death, for each year 2017 to 2025. The target probability in 2025 was calculated as the probability of premature death observed in 2010, multiplied by 0.75 to estimate a 25% reduction. The percentage reduction in the projected probability of premature death was calculated for 2025 compared to 2010, to estimate whether the target 25% reduction was met.

### Subgroup analysis

To assess whether premature mortality, projected age-specific mortality rates and the probability of death from the NCD4 in 2025 varied by age or sex, age-specific rates and ASMRs in 2016 were calculated for each NCD4 condition and by sex (male and female). The projected probability of premature death in 2025 was calculated for each NCD4 separately, and combined, and by sex.

### Sensitivity analyses

Two sensitivity analyses were conducted to assess the influence of key assumptions on the projection model.

First, we calculated the projected ASMRs and probability of premature death in 2025 using alternate population projection scenarios, series A and C, for Australia [13], reflecting projected changes in the Australian populations under an increase and decrease, respectively, in trends of migration, fertility and life expectancy.

Second, we used two different methods accounting for multiple causes of death to address the reliance of the analysis on the use of underlying cause of death, as specified by the WHO methodology. To account for multiple causes of death, associated causes of deaths listed in the deaths data were used to identify and recalculate the number of deaths in two ways: (1) counting deaths by any mention of an NCD4 as an underlying or associated cause and (2) counting deaths by assigning equal weighting to each cause contributing to the death, except duplicate mentions and ill-defined associated causes [16]. These alternate counts of the number of deaths were used to calculate the premature ASMR and probability of premature death in 2010–2016 and the projected premature ASMR and probability of premature death 2017-2015 as in the main analysis.

### Validation of projection estimates for 2017

Deaths in 2017 were not included in the main analysis due to lags in death registration, affecting about 4–7% of deaths in a given year [17].

Instead, the number of deaths estimated to occur in 2017 was estimated using the projection models. To check the validity of the projections for 2017, the observed rates for deaths registered in 2017 were compared to predicted rates for 2017 using a rate ratio. We defined a priori that a point estimate for the rate ratio for projected vs observed ASMR between 0.9 and 1.1 would be acceptable.

## Results

The study population in 2016, consisted of 12,155,608 adults resident in Australia aged 30–69 years, with 50.64% females (Table 2).

**Table 2:** Study population aged 30-69 years, deaths and proportional mortality in 2016, for cancer, cardiovascular disease (CVD), respiratory diseases and diabetes (NCD4)

	Population	% of total Australian population (all ages)	Number of deaths	Proportional mortality (%) by underlying cause of death					
				Cancer	CVD	Chronic respiratory diseases	Diabetes	NCD4 combined	Other causes
Persons	12,155,608	50.25	36,327	42.94	18.42	4.89	2.79	69.05	30.96
Males	6,000,459	24.81	22,501	38.64	21.25	4.39	2.92	67.20	32.80
Females	6,155,149	25.44	13,826	49.94	13.80	5.71	2.60	72.05	27.96

*Notes*: The proportional mortality was calculated at the national level as the percentage of total deaths within the study population

### Observed deaths, death rates and probability of dying

In 2016, there was a total of 158,426 deaths across the population aged 18 years and older, of which 105,128 (66.36%) were due to NCD4. Of the population aged 30–69 years, there were 36,327 deaths in 2016 (sex ratio of 1.63 of males to females), of which 25,082 (69.05%) were due to all NCD4 conditions combined. Most deaths (42.94%) were due to cancer, followed by CVD (18.42%) (Table 2). The number of people in the population, total number of deaths and proportion of the total mortality by each NCD4 condition is provided for each year (2010-2016) in Additional file 1: Table S1.

**Fig. 1:**
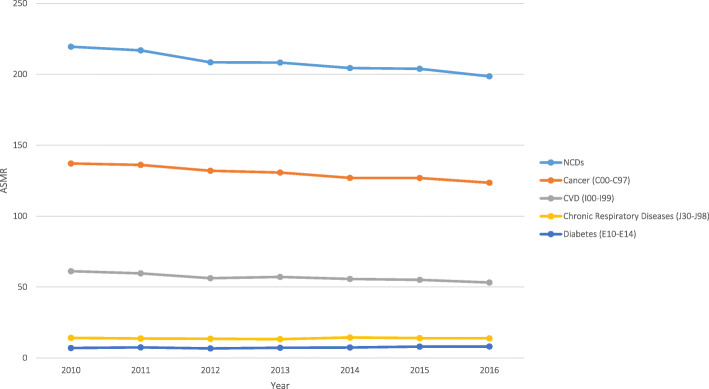
Age-standardised premature mortality rates for cancer, cardiovascular disease, respiratory diseases and diabetes combined in adults resident in Australia aged 30–69 years from 2010 to 2016. Notes: (1) The age-standardised mortality rates (ASMR) per 100,000 population were calculated at the national level and age-standardised using the 2011 Australian Estimated Resident Population of people aged 30–69. (2) The ASMRs for cancer, cardiovascular disease (CVD), chronic respiratory diseases and diabetes combined is displayed as non-communicable diseases (NCDs)

Over the period 2010–2016, 172,522 people aged 30–69 years died from all NCD4 conditions combined. The highest premature ASMRs were observed for cancer followed by CVD, chronic respiratory diseases and diabetes (Fig. 1). The premature ASMRs declined from 137.11 deaths per 100,000 in 2010 to 123.53 in 2016 for cancer (1.72% average annual decrease), 61.16 to 53.18 for CVD (2.28% average annual decrease), and 14.16 to 13.82 for chronic respiratory diseases (0.31% average annual decrease), contributing to a decline of 219.46 to 198.58 deaths per 100,000 for NCD4 conditions combined (Additional file 1: Table S2). Premature ASMR due to diabetes increased from 7.03 deaths per 100,000 in 2010 to 8.04 in 2016, with an average annual increase of 2.98% (Additional file 1: Table S2).

The probability of premature death from NCD4 declined from 9.84% in 2010 to 8.89% in 2016 (Table 3). This means a 30-year old living in Australia in 2016 had an 8.89% chance of dying from one of the NCD4s before reaching their 70th birthday, if the observed mortality rates for that year persisted at each age, and death did not occur due to another cause [18].

**Table 3:** Probability of dying aged 30–69 years in Australia, 2010–2016, from cancer, cardiovascular disease (CVD), respiratory diseases and diabetes (NCD4)

Year	Cancer	***Underlying cause of death***			
		CVD	Chronic respiratory diseases	Diabetes	NCD4 combined
**Probability of premature death (%)**					
2010	6.26	2.82	0.70	0.33	9.84
2011	6.20	2.73	0.68	0.36	9.71
2012	6.04	2.58	0.67	0.32	9.38
2013	5.98	2.62	0.66	0.34	9.36
2014	5.80	2.55	0.71	0.35	9.17
2015	5.77	2.52	0.69	0.37	9.12
2016	5.63	2.43	0.68	0.38	8.89
**Projected premature age-standardised mortality rate per 100,000 in 2025**					
2025	104.56	40.21	13.46	7.73	164.23
**Projected probability of premature death in 2025 (%)**					
2025	4.77	1.81	0.66	0.35	7.36
**Relative reduction in the projected probability of death compared to 2010 (%)**					
2025	–	–	–	–	25.16

*Notes*: (1) Rates represent deaths per 100,000, standardised to using the 2011 Australian Estimated Resident Population of people aged 30–69 years. (2) The probability of premature death due to cancer (C00–C97), cardiovascular disease (CVD) (I00–I99), chronic respiratory diseases (J30–J98) and diabetes (E10-E14) combined is displayed under non-communicable diseases (NCD4) combined

### Projected deaths, death rates and probability of dying

**Fig. 2:**
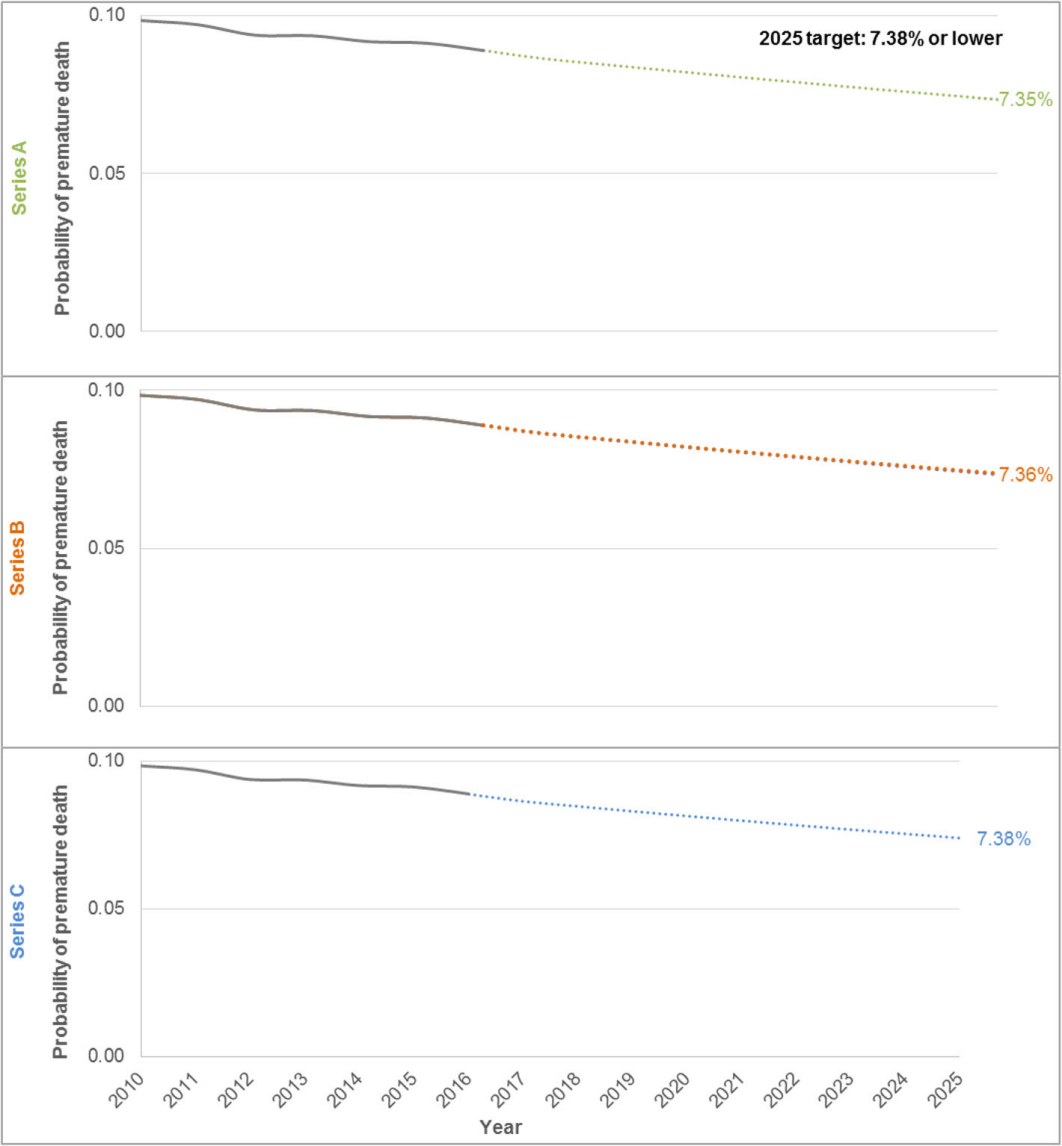
Probability of premature death due to cancer, cardiovascular disease, respiratory diseases and diabetes combined (NCD4), in Australia 2010–2025, by projected populations A, B, and C. Notes: (1) Solid lines indicate observed probability and non-solid/dashed lines indicate projected probability. (2) The projected probability of premature death in 2017–2025 was calculated using the data from 2006 to 2016 with the Australian Bureau of Statistics projected population series B which reflects the current trends in migration, fertility and life expectancy (main analysis, shown in orange). Sensitivity analyses using the alternative Australian Bureau of Statistics projected population series are shown in green (series A, reflecting an increase in migration, fertility and life expectancy) and purple (series C, showing a decrease in trends in migration, fertility and life expectancy). (3) Premature death refers to deaths in people between the exact ages of 30 and 69 years. (4) Deaths were included for NCD4 conditions which were identified using ICD-10 codes: cancer (C00–C97), cardiovascular disease (CVD) (I00–I99), chronic respiratory diseases (J30–J98) and diabetes (E10–E14)

Projected numbers of deaths and proportional mortality for each NCD4 condition for the years 2017 to 2025 are provided in Additional file 1: Table S1. The projected premature ASMR due to NCD4 conditions combined in 2025 was 164.23 per 100,000 (Table 3 and Additional file 1: Table S3). In 2025, the projected probability of premature death was 7.36% (Table 4 and Fig. 2) compared to 9.84% in 2010, representing a predicted relative reduction of 25.16% by 2025 (Table 3). Model fit was good (Additional file 1: Table S4).

**Table 4:** Probability of premature death for people aged 30–69 years, in Australia, 2005 projected to 2025, from cancer, cardiovascular disease, respiratory diseases and diabetes (NCD4)

Year	Probability of premature death due to NCD4 (%)					
	WHO estimate (24)			Australian estimate		
	Male	Female	Person	Male	Female	Person
2005^a^/2006 (2005 for WHO estimate)	13.90	8.80	11.40	13.12	8.26	10.71
2010	12.10	7.80	10.00	12.02	7.63	9.84
2015	11.30	7.30	9.30	11.10	7.15	9.12
2016	11.00	7.20	9.10	10.83	6.98	8.89
**2025 (target)**	**9.08**	**5.85**	**7.50**	**9.02**	**5.72**	**7.38**
**2025 (projected)**	–	–	–	**8.90**	**5.90**	**7.36**

*Notes*: (1) The probability of premature death due to non-communicable diseases (NCD4) represents the probability of death in persons aged 30–69 inclusive, in Australia, due to cancer (C00–C97), cardiovascular disease (CVD) (I00–I99), chronic respiratory diseases (J30–J98) and diabetes (E10–E14) combined. (2) The projected probability of premature death in 2025 was calculated using census and mortality data from 2006 to 2016 with the Australian Bureau of Statistics projected population series B which reflects the current trends in migration, fertility and life expectancy. (3) The WHO data can be viewed online [10]. (4) The probability of premature death in 2025 needed to meet the WHO 2025 target was calculated as the WHO probability of premature death in 2010 multiplied by 0.75 (to estimate a 25% reduction). (A) WHO estimate is given for 2005, and the Australian estimate was calculated for 2006

### Subgroup analysis

**Fig. 3:**
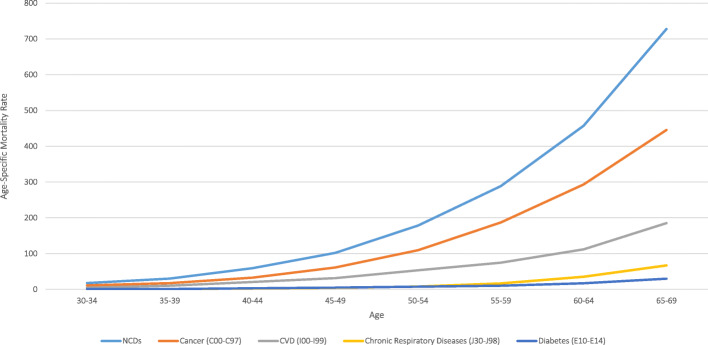
Age-specific mortality rates for cancer, cardiovascular disease (CVD), respiratory diseases and diabetes separately and combined in Australia, in 2016, by underlying cause of death across 5-year age groups. Notes: (1) The age-specific mortality rate per 100,000 population, for cancer, cardiovascular disease (CVD), chronic respiratory diseases and diabetes combined, is displayed as deaths caused by non-communicable diseases (NCDs).

**Fig. 4:**
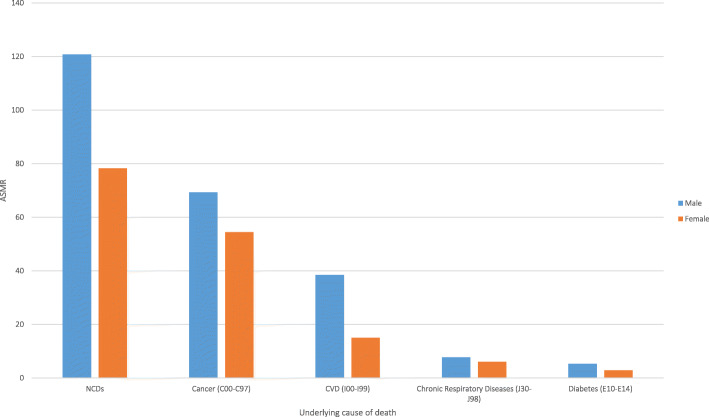
Age-standardised premature mortality rates by sex, for cancer, cardiovascular disease (CVD), respiratory diseases and diabetes in adults resident in Australia aged 30–69 years, in 2016. Notes: (1) The age-standardised mortality rates (ASMR) per 100,000 population were calculated at the national level and were age-standardised using the 2011 Australian Estimated Resident Population of people aged 30–69. (2) The age-standardised mortality rate for cancer, cardiovascular disease (CVD), chronic respiratory diseases and diabetes combined is displayed as deaths caused by non-communicable diseases (NCDs)

Within the population aged 30–69 years, rates of premature death increased with increasing age (Fig. 3) and premature ASMRs were higher in men compared to women for each NCD4 condition (Fig. 4). The same cause-specific patterns were observed by age and sex: the highest rates were for cancer followed by CVD, chronic respiratory diseases and diabetes. The greatest difference in ASMRs between males and females was for CVD (Fig. 4). The projected probability of premature death from NCD4 conditions in 2025 was higher in males than in females (8.90% vs. 5.90%; Additional file 2: Fig. S1).

### Sensitivity analyses

Using the different projected population scenarios, the probability and rate of premature mortality were slightly lower for series A (increased migration, fertility and life expectancy) and slightly higher for series C (decreased migration, fertility and life expectancy) compared with the results based on series B (current trends) (Fig. 2). However, this variation was small with all premature ASMRs for series A and C being within 1-unit death per 100,000 from series B, and the probability of premature death for series A and C being within 0.01% points from series B. The probability of premature death in 2025 was 7.35% for series A (increased migration, fertility and life expectancy) and 7.38% for series C (decreased migration, fertility and life expectancy) (Additional file 1: Table S5).

Patterns of results differed slightly from the main analysis when methods accounting for multiple causes of death were used (Additional file 2: Figs. S2-S4). Compared to the main results, the probability of premature death was higher using the method of any mention (11.32%) and lower using the equal weighting method (6.42%). This resulted in a relative reduction in the probability of death in 2025 compared to 2010 levels of 23.46% and 26.80%, respectively (Additional file 1: Table S6).

### Projection validation

There was little difference (1.37% deviation and a rate ratio of 1.01) between the observed and projected ASMRs for NCD4 conditions combined for 2017 (Additional file 1: Table S7).

## Discussion

Our study shows that if current trends in the mortality rate declines of NCD4 conditions continue, Australia can expect to see a reduction in the probability of premature death due to cancer, CVD, chronic respiratory diseases and diabetes, of around 25.16% by 2025. This suggests that meeting the WHO 25X25 goal is possible if actions to reduce NCDs continue.

Our large-scale study using whole-of-population data showed a decline in premature mortality in the Australian population due to cancer, CVD, chronic respiratory diseases and diabetes combined over the period 2010–2016, with age-standardised mortality rates declining by an average of 1.64% annually. Compared to 2010 levels, the probability of premature death is projected to reduce by around 25.16% in 2025.

Modelling by the NCD Countdown Collaborators relates to 2030 SDG targets, rather than 2025 ones and estimated decreases of > 15 and ≤ 20% in NCD4 conditions in Australia by 2030 compared to 2015 levels [9]. Australia has low premature NCD mortality by international standards, and this may contribute to a slower rate of decline in mortality. We expanded on previous analyses, by not only predicting premature mortality rates for NCDs by 2025 but also examining predicted rates for NCD4 conditions separately and by age and sex to provide information on points to target to further lower mortality rates. Taken together, our current analyses and those of the NCD Countdown Collaborators show that, if past trends continue, Australia might just meet the WHO 25X25 goal but is unlikely to meet the SDG goal of a 30% reduction by 2030 unless further declines in mortality are achieved.

The probability of premature death calculated for 2016 in the data (female 6.9%, male 10.8%) is very close to estimates from WHO and NCD Countdown Collaborators (female 7.1%, males 11%) [10, 11]. The observed decline in premature mortality due to NCD4 conditions combined over time is consistent with findings of a previous study that showed a reduction in the burden of disease from 2003 to 2015 [3] with age-standardised fatal burden (years of life lost) rates for cancer, CVD and respiratory diseases declining by a total of 6.1%, 11.3% and 0.5%, respectively. These findings [3] are consistent with estimates in our study of average annual decreases in ASMR of 1.72% for cancer, 2.28% for CVD and 0.32% for chronic respiratory diseases, from 2010 to 2016. While the previous study [3] reported no change in age-standardised years of life lost rate for diabetes over time, we observed a small increase in premature ASMR for diabetes. This is likely due to the changes in mortality coding associated with diabetes that were introduced in 2014 [19].

The subgroup analysis showed that while premature mortality rates declined in all groups, they were highest in males and older people. This is consistent with previously observed trends in Australia which have shown that males have had a persistently higher ASMR for all cancers combined compared to females from 1982 to 2016 and an increased fatal burden of chronic respiratory diseases in 2011, despite experiencing a similar total burden as females [20, 21]. Higher NCD mortality with increasing age was observed in our study and is well established in previous work including excess mortality rates of cancer from 1982 to 2003 and CVD mortality rates from 1980 to 2015 [22, 23].

This study is the first to predict the likelihood with which Australia will meet the WHO 25X25 goal, generating projections for the probability of premature mortality in 2025 in the context of this goal. The strengths of this study include the use of high-quality national-level data and the robustness of the results to different sensitivity analyses.

The large size and fine level of data in the Cause of Death Unit Record File allowed fine age-standardisation to compare rates over time. Future projections are sensitive to the data used to generate the projections as well as changes to population structures over time. To improve the stability of the projections, we used all available past data (2006–2016) to estimate future projections. We also undertook sensitivity analyses estimating the probability of death under two alternative projected population structures representing changes to fertility, migration and life expectancy. The projected probabilities of premature mortality were similar under these different scenarios, showing the results were robust to the assumptions of these models.

However, it is possible that the current COVID-19 pandemic will substantially impact trends in population growth, particularly migration, as Australia closed its borders to non-citizens between March 2020 and November 2021 [24]. This impact on the population structure may be considerable, given the role of net overseas migration as the largest source of population growth in Australia contributing 60.2% of population growth in 2019 [25]. Although we attempted to test for this using the alternative projected population structures provided by the Australian Bureau of statistics, these may not fully capture future changes resulting from the pandemic. Although mortality due to COVID-19 has been low in Australia so far, there may be increases in the number of preventable deaths over the next few years. For example, an increase in cancer deaths may result from reduced and delayed screening, diagnostic and treatment service utilisation which has been observed to coincide with the COVID-19 pandemic in Australia during 2020 [26]. Similar changes may be expected for other conditions such as CVD which rely on engagement with primary care for prevention and treatment.

The WHO 25X25 goal is based on reducing the probability of premature death from NCDs where these conditions are recorded as the underlying cause of death. However, death can often be attributed to multiple causes. In addition to examining the underlying cause of death in the main analysis, we used two alternative measures to account for each NCD4 condition’s contribution to death in the sensitivity analyses. The first which considered any mention of NCD4 conditions resulted in an increase in the estimated ASMRs, an increase in the probability of premature death during 2010–2016 and a decrease in the relative reduction of the probability of death by 2025 compared to what was observed in the main analysis. This was expected as capturing the prevalence at the death of all conditions allows for double counting of deaths, thereby inflating the numerator. We also used a multiple cause weighting approach where each cause contributing to each death is given an equal weighting relative to the total number of causes in the record. Using the multiple cause weighting approach, we projected around a 26% reduction in premature mortality from the NCD4 in 2025 compared to 2010.

In the analysis, several assumptions underpin the calculation of the probability of premature death and the projection model. Both use 5-year age groupings assuming that the subgroup trend is representative of each age contained within it.

Mortality analyses rely on the quality cause of death data. Australia has high coverage and applies international standard for coding causes and selecting underlying the cause of death. However, the Cause of Death Unit Record File is not immune from errors in certification of cause of death (e.g. by a doctor or coroner) that may arise from misdiagnosis and misreporting the role of disease in the causal sequence resulting in death [27].

To reduce premature mortality due to NCD4 conditions, for both the WHO 25X25 goal and the 2030 SDG goal, several studies have noted that country-specific action and targeting, based on recent trends in mortality, would be most effective [6, 9, 28]. Our study gives an overview of recent trends in premature mortality in Australia and identified areas where gains could be made to facilitate progress towards the WHO 25X25 goal. Overall, our results show that premature mortality rates were higher for males and older people (particularly from 60 to 69 years), with the greatest burden being due to cancer. Given the shared risk factors between NCDs and already low mortality rates in Australia, the biggest gains are likely to be made by targeting interventions to reduce common risk factors across the population, for example, in reducing smoking, and in targeting preventive interventions and treatments to high-risk groups by increasing screening and heath checks. Evaluating and comparing the expected public health impacts of these strategies is needed to further inform health policy.

## Conclusion

Premature mortality due to cancer, cardiovascular disease, respiratory diseases and diabetes has declined in Australia from 2010 to 2016. If past national trends continue, we predict relative reductions in the probability of premature mortality in Australia from all NCD4 conditions combined of around 25.16% in 2025 compared to 2010 levels. These findings highlight the importance of continuing and enhancing evidence-based policies and programmes targeting NCDs in Australia. This includes those targeting major and shared risk factors as well as improvements in coverage of disease screening and preventive treatments.

## Supplementary Information

**Additional file 1: Table S1.** Study population, number of deaths, and proportional mortality from NCD4 from 2010 to 2025. **Table S2.** Age-standardised rate and percentage change in rate of NCD4 mortality. **Table S3.** Projected age-standardised premature mortality rates from NCD4. **Table S4.** Model coefficients and Pearson’s goodness of fit test for Poisson regression models. **Table S5.** Sensitivity analysis using population series A, B and C. **Table S6**. Sensitivity analysis using multiple cause of death methods. **Table S7.** Validation of projection model for NCD4 mortality rates in 2017.

**Additional file 2: Figure S1.** Probability of premature death due to NCD4 in 2010-2025. **Figure S2.** Age-standardised premature mortality rates for NCD4 from 2010 to 2016, using multiple cause of death method equal weighting. **Figure S3.** Age-standardised premature mortality rates for NCD4 from 2010 to 2016, using multiple cause of death method any mention method. **Figure S4.** Probability of premature death due to NCD4 from 2010 to 2025, using multiple cause of death methods.

## Data Availability

The population datasets generated and/or analysed during the current study are available in the Australian Bureau of Statistics repository, http://stat.data.abs.gov.au/Index.aspx, https://www.abs.gov.au/AUSSTATS/abs@.nsf/DetailsPage/3222.02017%20(base)%20-%202066?OpenDocument. The death data that support the findings of this study are available from the Registrars of Births, Deaths and Marriages and the Coroners and the National Coronial Information System, but restrictions apply to the availability of these data, which were used under licence for the current study, and so are not publicly available. Data are however available from the authors upon reasonable request and with permission of the Registrars of Births, Deaths and Marriages and the Coroners and the National Coronial Information System.

## Abbreviations

ABS: Australian Bureau of Statistics
AIHW: Australian Institute of Health and Welfare
ASMR: Age-standardised mortality rate
CVD: Cardiovascular disease
NCD4: Cancer, cardiovascular disease, respiratory diseases and diabetes
NCDs: Non-communicable diseases
SDG: Sustainable Development Goal
WHO: World Health Organization
