# Supplementary material for: Observed and predicted premature mortality in Australia due to non-communicable diseases: a population-based study examining progress towards the WHO 25X25 goal
Source: BMC Med. 2022 Feb 10;20:57. doi: 10.1186/s12916-022-02253-z (PMC8830024; doi:10.1186/s12916-022-02253-z)
Supplement: Supplementary file 1 — Additional file 1: Table S1. Study population, number of deaths, and proportional mortality from NCD4 from 2010 to 2025. Table S2. Age-standardised rate and percentage change in rate of NCD4 mortality. Table S3. Projected age-standardised premature mortality rates from NCD4. Table S4. Model coefficients and Pearson’s goodness of fit test for Poisson regression models. Table S5. Sensitivity analysis using population series A, B and C. Table S6. Sensitivity analysis using multiple cause of death methods. Table S7. Validation of projection model for NCD4 mortality rates in 2017. [file 12916_2022_2253_MOESM1_ESM.docx]

**Observed and Predicted Premature Mortality in Australia due to Non-Communicable Diseases: A population-based study examining progress towards the WHO 25X25 goal**

Alison Wijnen, Karen Bishop, Grace Joshy, Yuehan Zhang, Emily Banks and Ellie Paige

**Additional File 1: Supplementary Tables**

**Table S1: Study population aged 30-69 years, number of deaths, and proportional mortality from cancer, cardiovascular disease (CVD), respiratory diseases and diabetes (NCD4) by sex, from 2010 to 2025**

| Year |  | Population | Number of deaths | Proportional mortality (%) by underlying cause of death | | | | | |
| --- | --- | --- | --- | --- | --- | --- | --- | --- | --- |
|  |  |  |  | Cancer | CVD | Respiratory disease | Diabetes | NCDs combined | Other causes |
| Observed | | | | | | | | | |
| 2010 | Persons | 11 062 414 | 33 500 | 44.72 | 19.96 | 4.60 | 2.30 | 71.58 | 28.42 |
|  | Male | 5 498 114 | 21 018 | 40.25 | 22.66 | 4.34 | 2.38 | 69.63 | 30.37 |
|  | Female | 5 564 300 | 12 482 | 52.25 | 15.42 | 5.04 | 2.16 | 74.87 | 25.13 |
| 2011 | Persons | 11 249 468 | 34 355 | 44.55 | 19.53 | 4.49 | 2.45 | 71.02 | 28.98 |
|  | Male | 5 589 457 | 21 535 | 40.00 | 22.74 | 3.99 | 2.46 | 69.18 | 30.82 |
|  | Female | 5 660 011 | 12 820 | 52.20 | 14.16 | 5.32 | 2.43 | 74.10 | 25.90 |
| 2012 | Persons | 11 449 805 | 34 151 | 44.75 | 19.06 | 4.63 | 2.27 | 70.72 | 29.29 |
|  | Male | 5 686 920 | 21 139 | 39.88 | 22.22 | 4.20 | 2.34 | 68.63 | 31.37 |
|  | Female | 5 762 885 | 13 012 | 52.67 | 13.93 | 5.33 | 2.17 | 74.11 | 25.89 |
| 2013 | Persons | 11 655 453 | 35 249 | 44.15 | 19.27 | 4.53 | 2.42 | 70.38 | 29.62 |
|  | Male | 5 785 068 | 21 950 | 39.98 | 22.30 | 4.25 | 2.51 | 69.03 | 30.97 |
|  | Female | 5 870 385 | 13 299 | 51.04 | 14.26 | 5.00 | 2.29 | 72.59 | 27.41 |
| 2014 | Persons | 11 823 409 | 36 076 | 42.77 | 18.69 | 4.94 | 2.47 | 68.86 | 31.14 |
|  | Male | 5 858 240 | 22 436 | 38.07 | 21.29 | 4.41 | 2.57 | 66.34 | 33.67 |
|  | Female | 5 965 169 | 13 640 | 50.51 | 14.40 | 5.81 | 2.30 | 73.02 | 26.98 |
| 2015 | Persons | 11 986 392 | 36 524 | 43.01 | 18.61 | 4.83 | 2.71 | 69.17 | 30.84 |
|  | Male | 5 928 750 | 22 759 | 38.29 | 21.52 | 4.39 | 2.91 | 66.99 | 33.01 |
|  | Female | 6 057 642 | 13 738 | 50.91 | 13.82 | 5.58 | 2.40 | 72.70 | 27.30 |
| 2016 | Persons | 12 155 608 | 36 327 | 42.94 | 18.42 | 4.89 | 2.79 | 69.05 | 30.96 |
|  | Male | 6 000 459 | 22 501 | 38.64 | 21.25 | 4.39 | 2.92 | 67.20 | 32.80 |
|  | Female | 6 155 149 | 13 826 | 49.94 | 13.80 | 5.71 | 2.60 | 72.05 | 27.96 |
| Projected | | | | | | | | | |
| 2017 | Persons | 12 313 800 | 36 042 | 42.90 | 17.85 | 4.95 | 2.66 | 68.26 | 31.74 |
|  | Male | 6 070 438 | 22 239 | 38.38 | 20.62 | 4.50 | 2.81 | 66.24 | 33.77 |
|  | Female | 6 243 362 | 13 775 | 50.21 | 13.34 | 5.68 | 2.41 | 71.53 | 28.47 |
| 2018 | Persons | 12 481 741 | 36 043 | 42.65 | 17.54 | 4.99 | 2.69 | 67.74 | 32.26 |
|  | Male | 6 146 596 | 22 185 | 38.14 | 20.28 | 4.55 | 2.86 | 65.69 | 34.31 |
|  | Female | 6 335 145 | 13 823 | 49.93 | 13.11 | 5.72 | 2.42 | 71.02 | 28.98 |
| 2019 | Persons | 12 661 623 | 36 094 | 42.41 | 17.23 | 5.04 | 2.72 | 67.22 | 32.78 |
|  | Male | 6 229 174 | 21 669 | 38.77 | 20.39 | 4.71 | 2.97 | 66.65 | 33.35 |
|  | Female | 6 432 449 | 13 888 | 49.64 | 12.87 | 5.78 | 2.43 | 70.51 | 29.49 |
| 2020 | Persons | 12 848 926 | 36 122 | 42.15 | 16.92 | 5.09 | 2.75 | 66.68 | 33.32 |
|  | Male | 6 316 768 | 22 149 | 37.64 | 19.59 | 4.65 | 2.95 | 64.59 | 35.41 |
|  | Female | 6 532 158 | 13 936 | 49.34 | 12.65 | 5.83 | 2.45 | 69.98 | 30.02 |
| 2021 | Persons | 13 033 312 | 36 183 | 41.89 | 16.62 | 5.14 | 2.79 | 66.15 | 33.85 |
|  | Male | 6 403 684 | 22 158 | 37.39 | 19.24 | 4.70 | 3.01 | 64.05 | 35.96 |
|  | Female | 6 629 628 | 13 994 | 49.04 | 12.43 | 5.88 | 2.48 | 69.45 | 30.55 |
| 2022 | Persons | 13 217 345 | 36 245 | 41.63 | 16.32 | 5.19 | 2.83 | 65.62 | 34.39 |
|  | Male | 6 491 628 | 22 169 | 37.14 | 18.91 | 4.75 | 3.06 | 63.49 | 36.51 |
|  | Female | 6 725 717 | 14 051 | 48.73 | 12.23 | 5.94 | 2.51 | 68.92 | 31.08 |
| 2023 | Persons | 13 401 150 | 36 288 | 41.36 | 16.03 | 5.24 | 2.87 | 65.07 | 34.93 |
|  | Male | 6 581 013 | 22 176 | 36.87 | 18.57 | 4.80 | 3.11 | 62.92 | 37.08 |
|  | Female | 6 820 137 | 14 098 | 48.41 | 12.01 | 6.00 | 2.53 | 68.38 | 31.62 |
| 2024 | Persons | 13 592 177 | 36 342 | 41.08 | 15.74 | 5.29 | 2.91 | 64.51 | 35.49 |
|  | Male | 6 675 611 | 22 200 | 36.61 | 18.24 | 4.86 | 3.17 | 62.36 | 37.64 |
|  | Female | 6 916 566 | 14 141 | 48.09 | 11.80 | 6.06 | 2.55 | 67.82 | 32.18 |
| 2025 | Persons | 13 782 560 | 36 361 | 40.78 | 15.45 | 5.34 | 2.94 | 63.93 | 36.07 |
|  | Male | 6 770 770 | 22 199 | 36.32 | 17.92 | 4.91 | 3.22 | 61.76 | 38.24 |
|  | Female | 7 011 790 | 14 173 | 47.77 | 11.60 | 6.11 | 2.58 | 67.26 | 32.74 |

**Table S2: Age-standardised rate and percentage change in rate of NCDs mortality in adults resident in Australia aged 30-69, from 2010 to 2016**

|  |  |  | *Underlying cause of death* | | | |
| --- | --- | --- | --- | --- | --- | --- |
|  |  | Cancer | CVD | Chronic Respiratory Diseases | Diabetes | NCDs combined |
| National age-standardised premature mortality rate, per 100 000 | 2010 | 137.11 | 61.16 | 14.16 | 7.03 | 219.46 |
|  | 2011 | 136.06 | 59.66 | 13.70 | 7.47 | 216.88 |
|  | 2012 | 132.01 | 56.24 | 13.55 | 6.68 | 208.49 |
|  | 2013 | 130.74 | 57.14 | 13.24 | 7.16 | 208.28 |
|  | 2014 | 126.96 | 55.67 | 14.45 | 7.31 | 204.39 |
|  | 2015 | 126.88 | 55.05 | 13.97 | 8.01 | 203.92 |
|  | 2016 | 123.53 | 53.18 | 13.82 | 8.04 | 198.58 |
|  |  |  |  |  |  |  |
| Annual percentage change in age-standardised premature mortality rate compared to previous year | 2011 | -0.76 | -2.46 | -3.28 | 6.18 | -1.18 |
|  | 2012 | -2.97 | -5.73 | -1.07 | -10.48 | -3.87 |
|  | 2013 | -0.97 | 1.60 | -2.33 | 7.15 | -0.10 |
|  | 2014 | -2.89 | -2.58 | 9.16 | 2.11 | -1.87 |
|  | 2015 | -0.06 | -1.10 | -3.31 | 9.55 | -0.23 |
|  | 2016 | -2.64 | -3.40 | -1.09 | 0.36 | -2.62 |
| Average annual percentage change in age-standardised premature mortality rate 2010-2016 |  | -1.72 | -2.28 | -0.31 | 2.49 | -1.64 |

Notes: (1) Population numbers from 2010 to 2016 were based on Estimated Resident Population of people aged 30-69 years. Population numbers from 2017 to 2025 are based on Australian Bureau of Statistics projected population series B, which reflects current trends in migration, fertility and life expectancy. (2) The mortality rates were calculated at the national level and age-standardised using the 2011 Australian Estimated Resident Population of people aged 30-69 years. (3) The age-standardised mortality rate for cancer (C00-C97), cardiovascular disease (CVD) (I00-I99), chronic respiratory diseases (J30-J98) and diabetes (E10-E14) combined is displayed as deaths caused by non-communicable diseases (NCD4) combined.

**Table S3: Projected age-standardised premature mortality rates from cancer, cardiovascular disease (CVD), respiratory diseases and diabetes (NCD4) in Australia, in each year 2017 to 2025**

| Year |  | *Underlying cause of death*  *(per 100 000 population)* | | | |
| --- | --- | --- | --- | --- | --- |
|  | Cancer | CVD | Chronic Respiratory Diseases | Diabetes | NCDs combined |
| 2017 | 121.09 | 50.64 | 13.71 | 7.52 | 192.69 |
| 2018 | 118.85 | 49.15 | 13.67 | 7.53 | 188.81 |
| 2019 | 116.67 | 47.72 | 13.63 | 7.54 | 185.02 |
| 2020 | 114.54 | 46.35 | 13.59 | 7.56 | 181.34 |
| 2021 | 112.46 | 45.03 | 13.56 | 7.58 | 177.75 |
| 2022 | 110.42 | 43.76 | 13.53 | 7.61 | 174.25 |
| 2023 | 108.42 | 42.53 | 13.50 | 7.64 | 170.83 |
| 2024 | 106.47 | 41.35 | 13.48 | 7.68 | 167.50 |
| 2025 | 104.56 | 40.21 | 13.46 | 7.73 | 164.23 |

Notes: (1) The projected premature mortality rates were calculated with Australian Bureau of Statistics projected population series B, which reflects current trends in migration, fertility and life expectancy. (2) The rates were age-standardised using the 2011 Australian Estimated Resident Population of people aged 30-69. (3) The age-standardised mortality rate for cancer (C00-C97), cardiovascular disease (CVD) (I00-I99), chronic respiratory diseases (J30-J98) and diabetes (E10-E14) combined is displayed as deaths caused by non-communicable diseases (NCD4) combined.

**Table S4: Model coefficients and Pearson’s goodness of fit test for 80 Poisson regression models predicting deaths from cancer, cardiovascular disease (CVD), respiratory diseases and diabetes (NCD4) combined**

|  | Model | | IRR* (95% CI) | Pearson’s value | Chi-squared P-value |
| --- | --- | --- | --- | --- | --- |
| NCD | Sex | Age |  | - | - |
| NCD4 combined | Male | 30-34 | 0.97 (0.95 - 0.98) | 8.59 | 0.48 |
|  |  | 35-39 | 0.98 (0.97 - 0.99) | 8.88 | 0.45 |
|  |  | 40-44 | 0.98 (0.98 - 0.99) | 20.13 | 0.02 |
|  |  | 45-49 | 0.98 (0.98 - 0.99) | 14.86 | 0.09 |
|  |  | 50-54 | 0.99 (0.98 - 0.99) | 13.57 | 0.14 |
|  |  | 55-59 | 0.99 (0.98 - 0.99) | 18.17 | 0.03 |
|  |  | 60-64 | 0.98 (0.98 - 0.98) | 12.15 | 0.21 |
|  |  | 65-69 | 0.97 (0.97 - 0.97) | 12.08 | 0.21 |
|  | Female | 30-34 | 1.00 (0.98 - 1.01) | 19.20 | 0.02 |
|  |  | 35-39 | 0.99 (0.97 - 1.00) | 6.10 | 0.73 |
|  |  | 40-44 | 0.99 (0.98 - 1.00) | 8.91 | 0.45 |
|  |  | 45-49 | 0.98 (0.98 - 0.99) | 7.87 | 0.55 |
|  |  | 50-54 | 0.99 (0.98 - 0.99) | 7.17 | 0.62 |
|  |  | 55-59 | 0.99 (0.98 - 0.99) | 2.28 | 0.99 |
|  |  | 60-64 | 0.98 (0.97 - 0.98) | 8.47 | 0.49 |
|  |  | 65-69 | 0.98 (0.98 - 0.98) | 10.85 | 0.29 |
| Cancer | Male | 30-34 | 0.99 (0.97 - 1.01) | 9.49 | 0.39 |
|  |  | 35-39 | 0.98 (0.97 - 1.00) | 2.43 | 0.98 |
|  |  | 40-44 | 0.98 (0.97 - 1.00) | 12.75 | 0.17 |
|  |  | 45-49 | 0.98 (0.97 - 0.99) | 15.64 | 0.07 |
|  |  | 50-54 | 0.98 (0.98 - 0.99) | 5.37 | 0.80 |
|  |  | 55-59 | 0.99 (0.98 - 0.99) | 7.30 | 0.61 |
|  |  | 60-64 | 0.98 (0.98 - 0.99) | 8.14 | 0.52 |
|  |  | 65-69 | 0.98 (0.97 - 0.98) | 6.46 | 0.69 |
|  | Female | 30-34 | 1.01 (0.99 - 1.04) | 14.65 | 0.10 |
|  |  | 35-39 | 0.99 (0.97 - 1.00) | 9.22 | 0.42 |
|  |  | 40-44 | 0.98 (0.97 - 0.99) | 8.37 | 0.50 |
|  |  | 45-49 | 0.98 (0.97 - 0.99) | 9.87 | 0.36 |
|  |  | 50-54 | 0.98 (0.98 - 0.99) | 10.27 | 0.33 |
|  |  | 55-59 | 0.98 (0.98 - 0.99) | 4.46 | 0.88 |
|  |  | 60-64 | 0.98 (0.97 - 0.98) | 8.30 | 0.50 |
|  |  | 65-69 | 0.98 (0.98 - 0.99) | 3.17 | 0.96 |
| CVD | Male | 30-34 | 0.94 (0.92 - 0.96) | 9.19 | 0.42 |
|  |  | 35-39 | 0.98 (0.96 - 0.99) | 9.69 | 0.38 |
|  |  | 40-44 | 0.98 (0.97 - 0.99) | 8.34 | 0.50 |
|  |  | 45-49 | 0.98 (0.97 - 0.99) | 9.93 | 0.36 |
|  |  | 50-54 | 0.98 (0.97 - 0.99) | 14.50 | 0.11 |
|  |  | 55-59 | 0.98 (0.97 - 0.99) | 13.34 | 0.15 |
|  |  | 60-64 | 0.97 (0.96 - 0.98) | 9.77 | 0.37 |
|  |  | 65-69 | 0.96 (0.96 - 0.96) | 15.50 | 0.08 |
|  | Female | 30-34 | 0.95 (0.92 - 0.98) | 6.97 | 0.64 |
|  |  | 35-39 | 0.98 (0.96 - 1.01) | 4.55 | 0.87 |
|  |  | 40-44 | 1 (0.98 - 1.02) | 16.80 | 0.05 |
|  |  | 45-49 | 0.98 (0.97 - 1.00) | 7.43 | 0.59 |
|  |  | 50-54 | 0.99 (0.98 - 1.00) | 12.01 | 0.21 |
|  |  | 55-59 | 0.99 (0.98 - 1.00) | 13.62 | 0.14 |
|  |  | 60-64 | 0.96 (0.96 - 0.97) | 9.73 | 0.37 |
|  |  | 65-69 | 0.96 (0.95 - 0.96) | 22.67 | 0.01 |
| Chronic Respiratory Diseases | Male | 30-34 | 0.97 (0.91 - 1.03) | 13.37 | 0.15 |
|  |  | 35-39 | 1.00 (0.95 - 1.05) | 15.12 | 0.09 |
|  |  | 40-44 | 0.98 (0.94 - 1.03) | 2.54 | 0.98 |
|  |  | 45-49 | 1.03 (1.00 - 1.07) | 9.64 | 0.38 |
|  |  | 50-54 | 1.01 (0.98 - 1.03) | 14.59 | 0.10 |
|  |  | 55-59 | 1.02 (1.00 - 1.04) | 3.35 | 0.95 |
|  |  | 60-64 | 0.99 (0.98 - 1.01) | 3.39 | 0.95 |
|  |  | 65-69 | 0.99 (0.98 - 1.00) | 9.73 | 0.37 |
|  | Female | 30-34 | 0.96 (0.89 - 1.03) | 14.00 | 0.12 |
|  |  | 35-39 | 0.98 (0.92 - 1.04) | 18.46 | 0.03 |
|  |  | 40-44 | 1.02 (0.97 - 1.07) | 18.25 | 0.03 |
|  |  | 45-49 | 0.99 (0.96 - 1.03) | 5.69 | 0.77 |
|  |  | 50-54 | 1.02 (0.99 - 1.04) | 9.26 | 0.41 |
|  |  | 55-59 | 0.99 (0.97 - 1.01) | 20.37 | 0.02 |
|  |  | 60-64 | 0.98 (0.97 - 1.00) | 15.18 | 0.09 |
|  |  | 65-69 | 1.00 (0.99 - 1.01) | 7.59 | 0.58 |
| Diabetes | Male | 30-34 | 1.02 (0.95 - 1.10) | 11.93 | 0.22 |
|  |  | 35-39 | 1.01 (0.95 - 1.07) | 6.40 | 0.70 |
|  |  | 40-44 | 1.02 (0.98 - 1.07) | 13.27 | 0.15 |
|  |  | 45-49 | 1.03 (1.00 - 1.07) | 14.63 | 0.10 |
|  |  | 50-54 | 1.04 (1.01 - 1.07) | 11.83 | 0.22 |
|  |  | 55-59 | 1.01 (0.99 - 1.03) | 20.55 | 0.01 |
|  |  | 60-64 | 0.99 (0.98 - 1.01) | 7.38 | 0.60 |
|  |  | 65-69 | 0.99 (0.97 - 1.00) | 17.10 | 0.05 |
|  | Female | 30-34 | 1.04 (0.96 - 1.13) | 17.49 | 0.04 |
|  |  | 35-39 | 0.98 (0.92 - 1.06) | 10.07 | 0.34 |
|  |  | 40-44 | 1.07 (1.01 - 1.12) | 4.48 | 0.88 |
|  |  | 45-49 | 0.99 (0.95 - 1.03) | 5.66 | 0.77 |
|  |  | 50-54 | 1.03 (1.00 - 1.07) | 3.70 | 0.93 |
|  |  | 55-59 | 0.99 (0.97 - 1.02) | 3.86 | 0.92 |
|  |  | 60-64 | 1.00 (0.98 - 1.02) | 8.44 | 0.49 |
|  |  | 65-69 | 0.96 (0.94 - 0.98) | 11.92 | 0.22 |

Notes: (1) The 80 models shown in the table predicted the number of deaths due to cancer, cardiovascular disease (CVD), chronic respiratory diseases and diabetes, separately and combined. (2) A non-statistically significant Chi-squared p-value (p > 0.05) indicates goodness of fit for the model i.e. there is not a statistically significant difference between the observed and modelled data. (3) IRR is the incidence rate ratio from Poisson regression models, indicating the ratio of the number of deaths in the year to the previous year. A statistically significant IRR less than 1 indicates a decrease in the number of death in the relevant year compared to the last year; a statistically significant IRR more than 1 indicates an increase in the number of deaths in the relevant year compared to the last year.

**Table S5: Sensitivity analysis using population series A, B and C, Australia**

|  |  |  | *Underlying cause of death* | | | |
| --- | --- | --- | --- | --- | --- | --- |
|  | ABS population projection series | Cancer | CVD | Chronic respiratory diseases | Diabetes | NCD4 |
| Projected premature ASMR in 2025 per 100 000 | A | 104.32 | 40.11 | 13.43 | 7.71 | 163.85 |
|  | B | 104.56 | 40.21 | 13.46 | 7.73 | 164.23 |
|  | C | 104.75 | 40.29 | 13.48 | 7.75 | 164.54 |
| Projected probability of dying 2025 (%) | A | 4.76 | 1.81 | 0.66 | 0.35 | 7.35 |
|  | B | 4.77 | 1.81 | 0.66 | 0.35 | 7.36 |
|  | C | 4.78 | 1.81 | 0.66 | 0.35 | 7.38 |
|  |  |  |  |  |  |  |
| Relative reduction in projected probability of death compared to 2010 (%) | A | 23.90 | 35.96 | 6.28 | -5.78 | 25.32 |
|  | B | 23.74 | 35.82 | 6.13 | -6.08 | 25.16 |
|  | C | 23.61 | 35.71 | 5.99 | -6.38 | 25.04 |

Notes: (1) The projected premature mortality rates and probability of premature death were calculated with Australian Bureau of Statistics projected population series A, B or C. These series correspond to scenarios of trends in migration, fertility and life expectancy. Series A corresponds to an increase in these trends, series B corresponds to current trends and series C corresponds to a decrease in these trends. (2) The premature age-standardised mortality rates (ASMR) were age-standardised using the 2011 Australian Estimated Resident Population of people aged 30-69. (4) The probability of premature death due to NCD4 represents the probability of death in persons aged 30-69 years, due to cancer (C00-C97), cardiovascular disease (CVD) (I00-I99), chronic respiratory diseases (J30-J98) and diabetes (E10-E14) combined.

**Table S6: Sensitivity analysis using multiple cause of death methods**

| Multiple cause of death method of counting deaths | Year | **Probability of premature death (%)** | | | | | **Age standardised mortality rate (per 100 000)** | | | | |
| --- | --- | --- | --- | --- | --- | --- | --- | --- | --- | --- | --- |
|  |  | Cancer | CVD | Chronic Respiratory Diseases | Diabetes | NCD4 | Cancer | CVD | Chronic Respiratory Diseases | Diabetes | NCD4 |
| **Any mention** | 2010 | 6.58 | 5.37 | 2.41 | 1.23 | 14.79 | 144.04 | 116.76 | 50.32 | 25.56 | 336.68 |
|  | 2025 projected | 4.92 | 3.92 | 1.95 | 1.11 | 11.32 | 107.34 | 84.38 | 41.30 | 23.79 | 255.89 |
|  | 2025 target | 4.93 | 4.03 | 1.80 | 0.92 | 11.09 | - | - | - | - | - |
|  | 2025  Relative reduction in projected probability of death compared to 2010 | 25.23% | 27.00% | 19.09% | 9.76% | 23.46% | - | - | - | - | - |
| **Equal weighting** | 2010 | 4.84 | 2.97 | 0.83 | 0.37 | 8.77 | 105.76 | 64.1 | 17.02 | 7.68 | 194.56 |
|  | 2025 projected | 3.64 | 1.90 | 0.72 | 0.31 | 6.42 | 79.45 | 41.69 | 15.00 | 6.69 | 142.01 |
|  | 2025 target | 3.39 | 2.22 | 0.62 | 0.28 | 6.58 | - | - | - | - | - |
|  | 2025  Relative reduction in projected probability of death compared to 2010 | 24.79% | 36.03% | 13.25% | 16.22% | 26.80% | - | - | - | - | - |

Notes: (1) Multiple causes of deaths methods were used for counting causes of deaths by any mention or weighting each cause. (2) The projected probability of premature death in 2025 was calculated using data from 2010-2016, with Australian Bureau of Statistics projected population series B which reflects current trends in migration, fertility and life expectancy. (3) The age-standardised mortality rates (ASMR) per 100 000 population were calculated at the national level and age-standardised using the 2011 Australian Estimated Resident Population of people aged 30-69. (4) The ASMRs for cancer, cardiovascular disease (CVD), chronic respiratory diseases and diabetes combined is displayed as NCD4. (5) Premature death refers to deaths in people between the exact age 30 and 69 years. (5) Deaths were identified using ICD-10 codes: cancer (C00-C97), cardiovascular disease (CVD) (I00-I99), chronic respiratory diseases (J30-J98) and diabetes (E10-E14).

**Table S7: Validation of projection model, observed and projected mortality rates for NCDs separately and combined, in Australia, in 2017**

|  |  |  | *Underlying cause of death* | | | |
| --- | --- | --- | --- | --- | --- | --- |
|  |  | Cancer | CVD | Chronic Respiratory Diseases | Diabetes | NCD4 |
| Premature ASMR in 2017, per 100 000 | Observed | 117.58 | 50.04 | 13.7 | 7.77 | 190.09 |
|  | Projected | 121.09 | 50.64 | 13.71 | 7.52 | 192.69 |
| Rate ratio | - | 1.03 | 1.01 | 1.00 | 0.97 | 1.01 |
| Absolute difference | - | 3.51 | 0.60 | 0.01 | -0.25 | 2.60 |
| Percent difference | - | 2.99% | 1.20% | 0.07% | -3.22% | 1.37% |

Notes: (1) The observed premature mortality rate in 2017 was determined using deaths that were registered in 2017. (2) The projected premature mortality rates were calculated using Cause of Death Unit Record File data from 2010 - 2016 with Australian Bureau of Statistics projected population series B which reflects current trends migration, fertility and life expectancy. (3) The premature age-standardised mortality rates (ASMR) were age-standardised using the 2011 Australian Estimated Resident Population of people aged 30-69. (4) The age-standardised mortality rate for cancer (C00-C97), cardiovascular disease (CVD) (I00-I99), chronic respiratory diseases (J30-J98) and diabetes (E10-E14) combined is displayed as deaths caused by NCD4. (6) The rate ratio was calculated by taking the quotient of the projected rate and the observed rate. (7) The absolute difference is the difference between the projected and the observed rates. (8) The percent difference is the absolute difference expressed as a percentage of the average between the observed and projected rates.
