# Supplementary material for: Observed and predicted premature mortality in Australia due to non-communicable diseases: a population-based study examining progress towards the WHO 25X25 goal
Source: BMC Med. 2022 Feb 10;20:57. doi: 10.1186/s12916-022-02253-z (PMC8830024; doi:10.1186/s12916-022-02253-z)
Supplement: Supplementary file 2 — Additional file 2: Figure S1. Probability of premature death due to NCD4 in 2010-2025. Figure S2. Age-standardised premature mortality rates for NCD4 from 2010 to 2016, using multiple cause of death method equal weighting. Figure S3. Age-standardised premature mortality rates for NCD4 from 2010 to 2016, using multiple cause of death method any mention method. Figure S4. Probability of premature death due to NCD4 from 2010 to 2025, using multiple cause of death methods. [file 12916_2022_2253_MOESM2_ESM.docx]

**Observed and Predicted Premature Mortality in Australia due to Non-Communicable Diseases: A population-based study examining progress towards the WHO 25X25 goal**

Alison Wijnen, Karen Bishop, Grace Joshy, Yuehan Zhang, Emily Banks and Ellie Paige

**Additional File 2: Supplementary Figures**

**
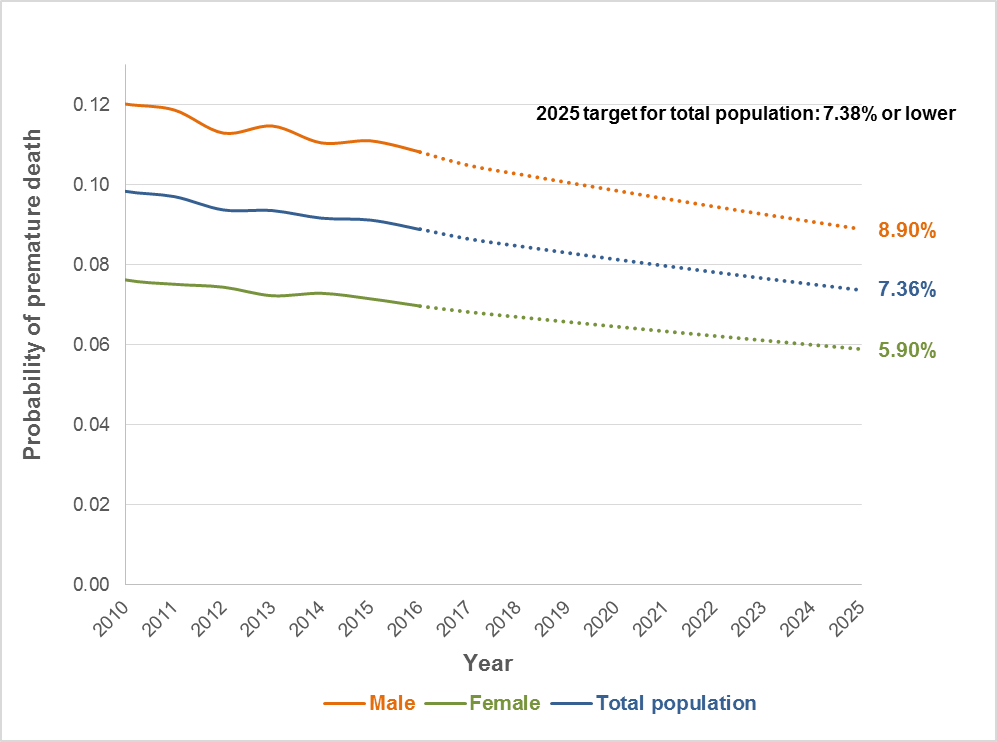
**

**Figure S1:** Probability of premature death due to cancer, cardiovascular disease, respiratory diseases and diabetes combined (NCD4), in Australia 2010-2025, by males and females

Notes: (1) Solid lines indicate observed probability and nonsolid/dashed lines indicate projected probability. (2) The projected probability of premature death in 2017-2025 was calculated using data from 2006-2016 with Australian Bureau of Statistics projected population series B which reflects current trends in migration, fertility and life expectancy. The middle (blue) line shows the probability of premature death for all people, the bottom (green) line shows results for females only, and the top (orange) line shows the results for males only. (3) Premature death refers to deaths in people between the exact age 30 and 69. (4) Deaths were included for NCD4 conditions which were identified using ICD-10 codes: cancer (C00-C97), cardiovascular disease (CVD) (I00-I99), chronic respiratory diseases (J30-J98) and diabetes (E10-E14).


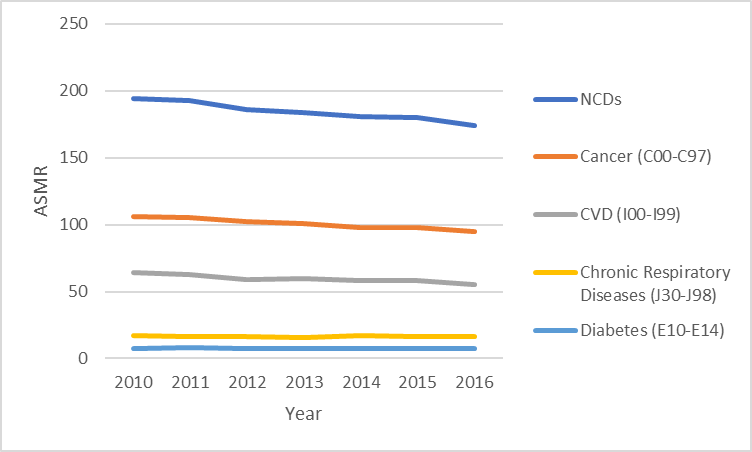


**Figure S2:** Age-standardised premature mortality rates (ASMR) for cancer, cardiovascular disease, respiratory diseases and diabetes combined in adults resident in Australia aged 30-69 years, from 2010 to 2016, using multiple cause of death method equal weighting

Notes: (1) The age-standardised mortality rates (ASMR) per 100 000 population were calculated at the national level and age-standardised using the 2011 Australian Estimated Resident Population of people aged 30-69. (2) The ASMRs for cancer, cardiovascular disease (CVD), chronic respiratory diseases and diabetes combined is displayed as non-communicable diseases (NCDs).


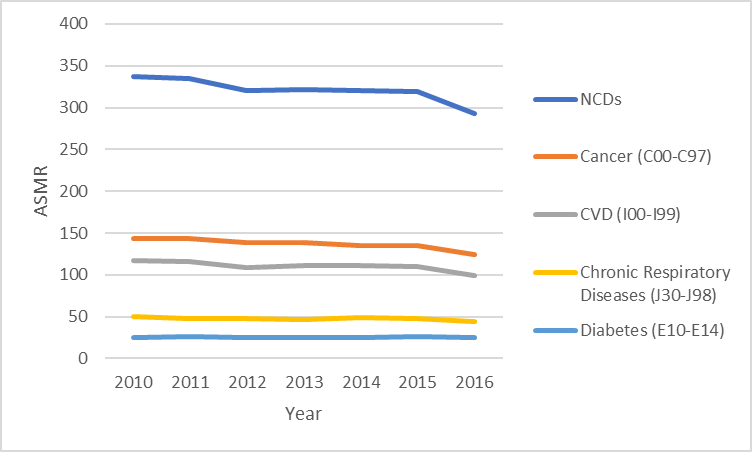


**Figure S3:** Age-standardised premature mortality rates (ASMR) cancer, cardiovascular disease, respiratory diseases and diabetes combined in adults resident in Australia aged 30-69 years, from 2010 to 2016, using multiple cause of death method any mention method

Notes: (1) The age-standardised mortality rates (ASMR) per 100 000 population were calculated at the national level and age-standardised using the 2011 Australian Estimated Resident Population of people aged 30-69. (2) The ASMRs for cancer, cardiovascular disease (CVD), chronic respiratory diseases and diabetes combined is displayed as non-communicable diseases (NCDs).

**Figure S4:** Probability of premature death due to cancer, cardiovascular disease, respiratory diseases and diabetes combined, in Australia, 2010 to 2025, using multiple cause of death methods

Notes: (1) Multiple causes of deaths methods were used for counting causes of deaths by any mention (blue) or weighting (orange). (2) Solid lines indicate observed probability and nonsolid/dashed lines indicate projected probability. (3) The projected probability of premature death in 2017-2025 was calculated using data from 2010-2016, with Australian Bureau of Statistics projected population series B which reflects current trends in migration, fertility and life expectancy. (4) Premature death refers to deaths in people between the exact age 30 and 69. (5) Deaths were identified using ICD-10 codes: cancer (C00-C97), cardiovascular disease (CVD) (I00-I99), chronic respiratory diseases (J30-J98) and diabetes (E10-E14).
